# Neighborhood Socioeconomic Deprivation Associated with Fat Mass and Weight Status in Youth

**DOI:** 10.3390/ijerph17176421

**Published:** 2020-09-03

**Authors:** Morgan Clennin, Asia Brown, Min Lian, Marsha Dowda, Natalie Colabianchi, Russell R. Pate

**Affiliations:** 1 Institute for Health Research, Kaiser Permanente Colorado, Aurora, CO 80014, USA; 2 Department of Exercise Science, University of South Carolina, Columbia, SC 29208, USA; mdowda@mailbox.sc.edu (M.D.); rpate@mailbox.sc.edu (R.R.P.); 3 Department of Psychology, University of South Carolina, Columbia, SC 29208, USA; asia@email.sc.edu; 4 Division of General Medical Sciences, Department of Medicine, Washington University School of Medicine, St. Louis, MO 63110, USA; mlian@wustl.edu; 5 School of Kinesiology & Institute for Social Research, University of Michigan, Ann Arbor, MI 48109, USA; colabian@umich.edu

**Keywords:** neighborhood deprivation, youth, obesity, physical activity, diet quality

## Abstract

(1) Background: Few studies have examined the relationship between neighborhood socioeconomic deprivation (SED) and weight-related outcomes in youth, controlling for weight-related behaviors. Hence, the purpose of this study was to examine the association between neighborhood SED, weight status, and fat mass in a diverse sample of youth, before and after controlling for physical activity and diet. (2) Methods: The sample included 828 youth from the Transitions and Activity Changes in Kids study. Neighborhood SED was expressed as an index score at the census tract of residence. Height, weight, and body composition were measured and used to calculate fat mass index (FMI) and weight status. Moderate-to-vigorous physical activity (MVPA) and sedentary behavior (min/h) were measured via accelerometry. Diet quality was assessed via the Block Food Screener for Kids. Multilevel regression models were employed to examine these relationships. (3) Results: Neighborhood SED was significantly associated with FMI and weight status before and after controlling for MVPA, sedentary behavior, and diet. Notably, youth residing in the most deprived neighborhoods had significantly higher FMI and were 30% more likely to be overweight/obese (OR = 1.30; 95% CI = 1.03–1.65). (4) Conclusions: Greater neighborhood SED was consistently and significantly associated with higher fat mass index and increased likelihood of overweight/obesity among youth.

## 1. Introduction

Over the past three decades, rates of childhood obesity have quadrupled in the U.S. [1,2]. According to the most recent national surveillance data (2015–2016), approximately 1 out of 5 U.S. youth are classified as obese [3]. Childhood obesity can lead to a host of negative health outcomes, including high blood pressure, high cholesterol, cardiovascular disease, insulin resistance, type 2 diabetes, respiratory problems, metabolic syndrome, and fatty liver disease [4,5,6,7]. Further, individuals who are classified as obese during childhood are more likely to be obese as adults, which is associated with a plethora of serious health conditions [5]. Despite recent evidence that rates of obesity have declined or begun to ‘level off’ among younger children (ages 2–11 years), obesity levels among older youth have continued to increase and disproportionately burden youth from lower socioeconomic and non-white racial/ethnic backgrounds [8]. This recent evidence suggests that public health efforts to address obesity have been effective among younger populations but have not effectively addressed factors that contribute to obesity among older youth [2]. One possible explanation for the trend in adolescent obesity rates is the increasing role that environmental factors play in health and health-related behaviors as youth age and become more independent and responsible for decisions that impact their health [9].

A growing body of evidence has consistently reported a positive association between neighborhood socioeconomic deprivation (SED) and numerous health outcomes, including weight status [10,11,12,13,14,15,16]. Across existing literature, a majority of studies have focused on the influence of neighborhood SED on broader health outcomes in adult populations [17]. Fewer studies have examined the influence of neighborhood SED on health outcomes and/or associated risk factors among youth. Of the studies that have examined this relationship, a majority support a significant relationship between neighborhood socioeconomic condition and youth weight-related outcomes, including BMI [18,19,20,21,22], weight status [9,23,24], and waist circumference [25]. Furthermore, several studies have noted that neighborhood SED attenuates the racial/SES disparities in body composition and weight status among youth [23,24].

While sufficient evidence exists to support a relationship between neighborhood SED and weight-related outcomes among youth, the underlying mechanisms that mediate this relationship have been relatively unexplored. Several health behaviors, including physical activity and diet, are well-established factors associated with weight status and body composition. However, few studies have examined the influence of physical activity and diet on the relationship between neighborhood SED and a measure of weight status and a measure of adiposity. Fewer studies have used objective and/or validated measures to assess these behaviors. Additionally, no study has examined the independent influence of the neighborhood SED on fat mass, a more accurate marker of body composition or adiposity than BMI percentiles. Therefore, the purpose of this study was to examine the associations between neighborhood SED, weight status, and fat mass in a diverse sample of youth, adjusting for physical activity and diet behaviors.

## 2. Materials and Methods

Data for this study were from the Transitions and Activity Changes in Kids (TRACK) study. TRACK was a multilevel study designed to examine the factors that influence physical activity as youth transition from elementary to middle school [26]. This cross-sectional study recruited 1090 5th-graders from 21 elementary schools in two urban South Carolina school districts in 2010. All students were invited to participate in this study during school assemblies (64% of students in one school district were recruited, 57% in the other). Written parental consent and child assent were obtained at enrollment and prior to assessment. Each enrolled participant then completed anthropometric measurements, received an accelerometer to measure physical activity, and completed a questionnaire. For this analysis, participants with missing data were excluded from the analytic sample. Participants excluded from the analytic sample (*n* = 262) were significantly younger, had poorer diet quality, were more likely to belong to a non-white race/ethnicity group, and resided in more deprived neighborhoods; no other differences were observed. The final analytic sample included 828 youth with complete data. This study was approved by the University of South Carolina’s Institutional Review Board (Pro00030375).

### 2.1. Weight-Related Outcomes

Weight status and fat mass index (FMI) were the primary outcome variables of interest for this study. To determine weight status, trained data collectors measured standing height to the nearest 0.1 cm using a portable stadiometer (SECA, Hamburg, Germany) and weight to the nearest 0.1 kg using a portable electronic scale (SECA, Hamburg, Germany). BMI was then calculated using the average of two measurements of height and weight via the standardized equation (kg/m^2^). Weight status was determined using age- and sex-specific body mass index (BMI) percentiles from the 2000 CDC growth charts and expressed as underweight/normal weight (<85th percentile) vs. overweight/obese (≥85th percentile) [27]. Due to small sample size, underweight (*n* = 8) was combined with normal weight BMI percentile category for all analyses. To calculate FMI, fat-free mass (i.e., resistance) was measured using total bioelectrical impedance (RJL QuantumIIBIA Systems, Clinton Township, MI, USA) in accordance with manufacturer protocol on the right side (hand and foot) [28]. Then fat mass was determined by subtracting fat-free mass from weight using the following equation: Fat Mass = Weight − [(3.474 + (0.459·Height^2^/Resistance) + (0.064·Weight)) ÷ (0.769 − (0.009·Age) − 0.016·Sex)], where 1 = male and 0 = female [28]. Finally, FMI was calculated by dividing fat mass by height (m^2^).

### 2.2. Neighborhood Socioeconomic Deprivation (SED)

Using the data from the 5 year estimates (2008–2012) of the American Community Survey (ACS), we developed a census tract-level SED index based on methods applied in previous studies [29]. Specifically, researchers have concluded that a composite index of neighborhood socioeconomic environment is a better measure than individual indicator variables and that the index should be based on the neighborhoods represented in the study area and at an appropriate scale. Referring to previous literature, we selected 21 census tract variables in 6 socioeconomic domains across the study region (Table S1). We assessed the structure of these variables across the study area using a principal component common factor analysis with the varimax rotation method. The largest proportion of the total variance for the data (35.9%) was explained by the first common factor. It included 12 variables with substantially greater factor loadings than those in other common factors (i.e., percentage of population with less than a high school education, percentage of working class, percentage of civilian labor force unemployed, percentage of households in poverty, percentage of female-headed households with dependent children, percentage of households with family income less than $30,000 per year, percentage of households with public assistance, percentage of households with no car, percentage of households with no phone, income disparity, percentage of population below the federal poverty line, and percentage of non-Hispanic African American population). The final composite SED index was computed using these 12 variables (Cronbach’s alpha = 0.93), which were standardized, weighted by scoring coefficients, and then summed together. The index was expressed as a continuous score at census tract level with higher values indicating greater deprivation.

### 2.3. Physical Activity

Physical activity was measured objectively using accelerometry (ActiGraph GT1M and GT3X models, Pensacola, FL, USA); only the vertical axis of the GT3X model was used in order to be comparable to the GT1M model. Previous studies demonstrated that accelerometers provide a reliable and validated assessment of objectively-measured physical activity levels among children and adolescents [30,31]. Briefly, each participant was instructed to wear an accelerometer on his or her right hip during waking hours for seven consecutive days, except while bathing, swimming, or sleeping. Data were collected and stored in 60 s epochs. All periods of non-wear time, defined as ≥60 min of consecutive zero activity counts, were set to missing [32]. Data for Sundays were excluded from the analytic dataset due to limited data availability. To be included in the analytic sample, at least two days with eight hours of accelerometer wear time each day were required. Missing values were then imputed using a sex-specific multiple imputation method via PROC MI in SAS (Version 9.3; SAS Institute, Inc., Cary, NC, USA). Validated age-specific thresholds were applied to accelerometer count data to determine activity levels [32]. Moderate-to-vigorous physical activity (MVPA) was defined as ≥2200 counts per minute [32,33]. Sedentary behavior was defined as <100 counts per minute [32,33]. Activity levels were expressed as average daily minutes of activity per hour of wear time.

### 2.4. Diet Quality

The Block Food Screener for Kids was used to assess students’ dietary intake across 41 unique food items over the past week [34]. During data collection, students completed the screener and identified each food item that they consumed in the past 7 days. For each food item selected, students then indicated the number of days that the item was consumed as well as the usual amount consumed. This tool has been validated in 10–17-year-olds via 24 h recalls [34]. To assess diet quality, a measure of diet quality based on an established protocol from the Healthy Eating Index (HEI)-2010 was constructed [35]. The score was calculated using a subset of food items on the screener that align with healthy dietary patterns (e.g., vegetable, fruit, whole grains, dairy, and protein) and adjusted by reported energy intake (i.e., per calorie consumed). Reported caloric intakes greater than 5000 kcal/day were deemed implausible and excluded from the analyses. The scores for the diet quality measure could range from zero to 50, with higher scores indicating better diet quality.

### 2.5. Student Characteristics

Participants reported their age, sex, and race/ethnicity via a student survey. Race and ethnicity groups were collapsed into four categories: non-Hispanic white, non-Hispanic black, Hispanic, and other (including multiracial). As part of the parent survey, a parent or guardian reported his or her highest level of education. Parent education was categorized into two groups (≤high school education; >high school education).

### 2.6. Statistical Analyses

Means and standard deviations were calculated for participant age, FMI, BMI, MVPA and sedentary behavior, diet quality, and neighborhood SED; frequencies and percentages were calculated for sex, race/ethnicity, parent education, and weight status. Univariate analyses were employed to examine distribution of all variables. To examine the relationship between neighborhood SED and weight-related outcomes, multilevel regression models accounted for the hierarchical structure of the data, with students nested within schools, were generated. Specifically, linear regression was used to examine the relationship between neighborhood SED and FMI and logistic regression was used to examine the relationship between neighborhood SED and weight status (overweight/obese = Y/N). First, the unadjusted associations between neighborhood SED and each outcome were examined (Model 1). Next age, sex, race/ethnicity, and parent education were added to the model to examine the adjusted relationship (Model 2). Finally, MVPA, sedentary behavior, and diet quality were added separately and then simultaneously to examine the fully adjusted relationship (Models 3–6). Model fit was assessed using maximum-likelihood estimation methods and Akaike’s Information Criterion (AIC), with lower values indicating improved model fit after the addition of independent variables of interest. An alpha level less than 0.05 was used to denote statistical significance for two-sided statistical tests. All model fit assumptions were examined and met. For ease of interpretation, the unadjusted and fully adjusted models were rerun using categorical expressions of neighborhood SED (quartiles) to produce least square means and odds ratios. All analyses were conducted in SAS 9.4 using the PROC MIXED procedure.

## 3. Results

### 3.1. Descriptives

Table 1 depicts the participant characteristics for the analytic sample. The mean age was 10.6 (±0.05) years and the sex distribution was approximately equal. The sample was racially and ethnically diverse. Nearly 60% of parents/guardians reported attending some college or obtaining a higher education degree beyond high school. Further, just over half of the sample was classified in the normal weight status category.

### 3.2. Multilevel Linear Regression Results

Table 2 presents results from multilevel linear regression models that assessed the relationship between neighborhood SED and FMI. Before and after controlling for demographic characteristics, FMI was significantly higher among students who resided in neighborhoods with greater deprivation (Models 1 and 2; *p* < 0.05). When added to the model independently, MVPA was negatively associated with FMI (Model 3; *p* < 0.001) and sedentary behavior (Model 4; *p* < 0.001) was positively associated with FMI; but diet quality was not (Model 5; *p* > 0.05). Additionally, the association between neighborhood SED and FMI remained after adjusting for sedentary behavior (Model 4; *p* < 0.05) and diet quality (Model 5; *p* < 0.05). However, the association was attenuated when MVPA was added to the model (Model 3; *p* > 0.05). The fully adjusted model indicated that neighborhood SED significantly associated with FMI even after controlling for MVPA, sedentary behavior, diet quality, and demographic characteristics (*p* < 0.05).

### 3.3. Multilevel Logistic Regression Results

Similar results were observed for the relationship between neighborhood SED and weight status (Table 3). Multilevel logistic regression indicated that, after adjusting for potential confounders, neighborhood SED was significantly associated with the odds of overweight/obesity among students. Specifically, the odds of overweight/obesity were 36% greater among youth living in more deprived neighborhood environment (Model 1: OR = 1.36, 95% CI = 1.10, 1.69). This association remained in the fully adjusted model after controlling for MVPA, sedentary behavior, diet quality, and demographic characteristics (Model 6: OR = 1.30, 95% CI = 1.03, 1.65). Additionally, greater minutes per hour of MVPA was also associated with decreased odds of overweight/obesity (OR = 0.80, 95% CI = 0.71–0.90). However, sedentary behavior and diet quality were not significantly associated with weight status in the fully adjusted model.

### 3.4. Adjusted Least Square Means

**Figure 1:**
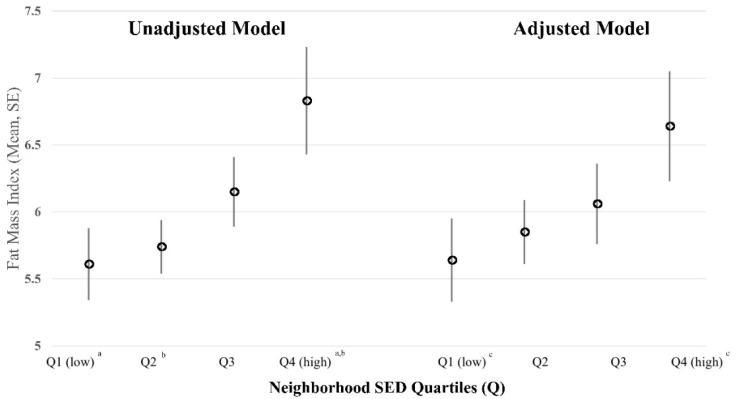
Unadjusted and adjusted least square means for fat mass index by neighborhood socioeconomic deprivation (SED) quartiles (Q). Model-derived estimates presented as least squared means and standard error (SE); unadjusted model accounted for participants clustered within schools; adjusted model controlled for age, sex, race/ethnicity, parent education, MVPA minutes per hour, sedentary minutes per hour, and diet quality; and accounted for participants clustered within schools. Neighborhood SED expressed as quartiles (Q), with low SED or more affluent neighborhood in the first quartile (Q1, low) and high SED or more deprived neighborhood in the fourth quartile (Q4, high). Superscript letters on *x* axis denote significant difference between least squared means across neighborhood SED quartiles, *p* < 0.05. ^a^ unadjusted model-derived estimate for fat mass index is significantly lower among youth residing in Q1 compared to youth residing in Q4; ^b^ unadjusted model derived estimate for fat mass index is significantly lower among youth residing in Q2 compared to youth residing in Q4; ^c^ adjusted model-derived estimate for fat mass index is significantly lower among youth residing in Q1 compared to youth residing in Q4.

To visually depict the relationship between neighborhood SED, FMI, and weight status, neighborhood SED index scores were categorized into quartiles and models were rerun. For FMI, least square means were generated. Figure 1 presents unadjusted and adjusted least square means for FMI across neighborhood SED quartiles (Q1–Q4). In both models, FMI was observed to increase as deprivation of the neighborhood increased from low SED (Q1) to high SED (Q4). In the fully adjusted model, FMI of youth who resided in the most socioeconomically deprived neighborhoods (Q4) was significantly greater compared to youth who resided in the most affluent neighborhoods (*p* < 0.05) (Table S2).

**Figure 2:**
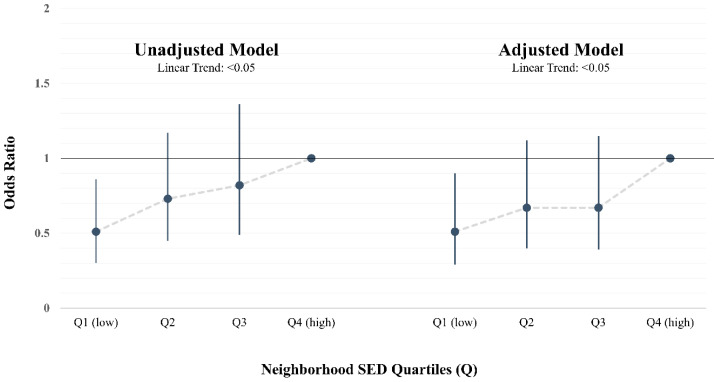
Unadjusted and adjusted odds of overweight/obesity by neighborhood socioeconomic deprivation (SED) quartiles. Unadjusted model accounted for participants clustered within schools. Adjusted model controlled for age, sex, race/ethnicity, parent education, MVPA minutes per hour, sedentary minutes per hour, and diet quality; and accounted for participants clustered within schools. Neighborhood SED expressed as quartiles (Q), with low SED or more affluent neighborhood in the first quartile (Q1, low) and high SED or more deprived neighborhood in the fourth quartile (Q4, high). Quartile 4 (Q4, high) serves as the reference group. Odds ratios depicted as dot, 95% confidence intervals depicted by vertical line, and linear trend in odds ratio across neighborhood SED quartiles depicted by dashed line.

To examine weight status, odds ratios for overweight/obesity were generated. Figure 2 presents the unadjusted and adjusted odds of overweight/obese status among 5th-grade students across neighborhood SED quartiles. Before and after adjusting for health behaviors and demographics, the odds of overweight/obesity were lower among students residing in neighborhoods with more favorable/affluent neighborhood socioeconomic environments (Q1). More specifically, the odds of overweight/obesity among youth residing in affluent neighborhoods (Q1) was observed to be nearly 50% less than the odds of overweight/obesity among youth residing in the most deprived neighborhoods (Q4). Further, the odds of overweight/obesity increased significantly across quartiles of neighborhood SED as deprivation increased (linear trend across quartile, *p* < 0.05) (Table S3).

## 4. Discussion

The key finding of the present study was a significant association between neighborhood SED and weight-related outcomes among a large and diverse sample of 5th-grade students in South Carolina. More specifically, the results demonstrated a consistent and positive association between neighborhood SED, fat mass, and the odds of overweight/obesity. With respect to fat mass, youth residing in more deprived neighborhood had significantly higher FMI compared to youth residing in more affluent neighborhoods. Further, the odds of overweight/obesity were half as likely among youth residing in affluent socioeconomic neighborhoods compared to youth residing in socioeconomically deprived neighborhoods. Notably, these relationships were observed independent of moderate-to-vigorous physical activity (MVPA), sedentary behavior, diet quality, and demographic characteristics. Our findings highlight the importance of the broader socioeconomic environment on weight-related outcomes in youth and suggest alternate pathways beyond health behaviors may play a vital role in influencing adiposity and weight status among this age group.

While BMI is a widely accepted measure of body fatness in epidemiology and clinical settings, it is a measure of total body weight relative to height—not a measure of adiposity [36]. BMI (or BMIz scores or BMI percentiles) does not distinguish between fat mass, muscle mass, or skeletal mass. This can lead to large errors in estimating adiposity (or fatness). Despite limitations of BMI in predicting adiposity among youth, it is still a primary metric used due to its unobtrusive and low-burden method of assessment. Future studies are needed to establish clinically significant cut points of fat mass among youth in order to promote widespread use of a true measure of adiposity in place of BMI.

With respect to weight status as determined by BMI percentile, our findings align with a majority of existing literature that has demonstrated a consistent and significant association between neighborhood SED and weight-related outcomes among youth [18,19,21,22,23,24,25]. Specifically, previous studies have reported significant associations between some indicators of neighborhood socioeconomic condition and youth BMI [18,19,20,21,22], weight status [9,23,24], and waist circumference and body mass [25]. However, few existing studies have examined these relationships while accounting for associated health behaviors such as physical activity.

The present study is one of the few to examine the relationship between neighborhood SED and weight-related outcomes while controlling for physical activity and dietary behaviors. However, the findings of our study differ from other studies that reported an attenuation of the relationship between neighborhood socioeconomic condition and weight-related outcomes after adjusting for individual-level health behaviors [25,37]. Nevill et al. (2015) reported a strong association between neighborhood deprivation and body mass among a sample of UK youth (10–16 yo) [25]. Notably, their observed relationship between neighborhood deprivation and body mass was non-significant after adjusting for cardiorespiratory fitness and self-reported physical activity simultaneously. The authors concluded that youth living in deprived neighborhoods were less physically fit and active [25]. Similarly, Slater et al. (2010) reported that lower neighborhood socioeconomic status, lower neighborhood safety, and higher neighborhood physical disorder were associated with increased BMI/obesity while higher neighborhood compactness was associated with lower BMI/obesity [37]. Contrary to our findings, the authors noted that neighborhood socioeconomic condition was associated with weight but not physical activity, which led them to conclude that an alternate causal pathway may better explain the relationship between neighborhood socioeconomic status and youth BMI/obesity [37]. The findings of the present study support this conclusion that alternate causal pathways may drive the relationship between neighborhood SED and weight-related outcomes.

Our results indirectly argue that health behaviors do not fully drive the relationship between neighborhood SED and weight. While the potential mediation was not formally tested due to the cross-sectional nature of this study, controlling for MVPA, sedentary behavior, and diet quality did not attenuate the observed relationship. Notably, MVPA was significantly associated with FMI and weight status in the final models while diet quality was not significantly associated. This non-significant association between diet quality and weight status may be due to the low reliability of dietary recall measures, particularly with non-white and underserved youth [38], or underreporting of fat and energy consumption among overweight youth [39]. Alternatively, physical activity and sedentary time may be better predictors of youth weight outcomes than diet. Longitudinal studies are needed in this area so that mediation analyses can be performed to disentangle complex causal relationships.

The present study adds to the limited literature examining this topic. However, some limitations should be noted. While we used an objective measure of physical activity, accelerometers are limited in their ability to capture some types of activities (i.e., non-weight-bearing and water-based activities) and do not provide contextual information (i.e., type and location). Such information might be useful in understanding the complex relationship between neighborhood SED and physical activity. With respect to neighborhood SED, the specific characteristics used were limited to those that were measured in existing data sources. As such, it is possible that some potential unmeasured confounders were not included in the analyses. Additionally, the use of residential census tracts is not a perfect measure of neighborhood; however, it has been widely used in previous studies [24,40]. Future studies should employ longitudinal designs to establish causality and how these relationships vary over time. Such studies should also explore the mediating roles of health behaviors such as physical activity on the relationship between neighborhood SED and weight-related outcomes as well as additional built and social environment characteristics.

## 5. Conclusions

In summary, we conclude that neighborhood SED matters with respect to weight-related outcomes among youth. Greater deprivation was associated with higher FMI and increased likelihood of overweight/obesity among youth. It is notable that the relationship between neighborhood SED, fat mass, and weight status was consistent and robust even after controlling for MVPA, sedentary behavior, and diet quality. This suggests that there may be other complex pathways and mechanisms that account for this complex relationship. Based on the findings and limitations of the present study, additional research is warranted to further disentangle these complete relationships. Given the strong and consistent association between neighborhood SED and multiple chronic health outcomes among adults, a better understanding of where and how this association emerges among younger populations is crucial to future public health efforts to address the growing burden of obesity and its related health outcomes.

## Figures and Tables

**Table 1:** Sample characteristics for Transitions and Activity Changes in Kids (TRACK) study participants (*n* = 828).

Participant Characteristics ^a^	Total Sample (*n* = 828)
Age (years)	10.6 (0.5)
Sex	
Male	45.3%
Female	54.7%
Race/Ethnicity	
Non-Hispanic White	39.1%
Non-Hispanic Black	33.6%
Hispanic	10.4%
Other	16.9%
Parent Education	
≤High School Education	42.3%
>High School Education	57.7%
Weight-Related Outcomes	
Fat Mass Index	5.9 (3.7)
Body Mass Index (BMI)	21.0 (4.7)
BMI Percentile	72.8 (26.9)
Weight Status	
Normal Weight/Underweight	53.1%
Overweight/Obese	46.9%
Physical Activity (PA)	
Moderate-to-Vigorous PA (Minutes/Hour) ^b^	2.8 (1.8)
Sedentary Behavior (Minutes/Hour) ^c^	32.1 (4.4)
Diet Quality	
Healthy Eating Index ^d^	30.1 (5.38)
Neighborhood Socioeconomic Deprivation ^e^	−0.19 (0.65)

^a^ Presented as the mean (standard deviation) unless otherwise denoted by percent, %; reported as percentage of column total. ^b^ Moderate-to-vigorous physical activity (MVPA) defined as ≥2200 counts per minute, as measured via accelerometry and expressed as average minutes of activity per hour of wear time. ^c^ Sedentary behavior defined as <100 counts per minute, as measured via accelerometry and expressed as average minutes of activity per hour of wear time. ^d^ The Heathy Eating Index (HEI) was constructed using dietary data from the Block Food Screener for Kids. Scores range from 0 to 50, with higher scores indicating better diet quality. ^e^ Index score was calculated using data from the American Community Survey 5-year estimates for the period 2008–2012. Neighborhood defined as census tract corresponding to participant’s home address. The index has a mean of zero and standard deviation of 1.

**Table 2:** Multilevel linear regression: fat mass index (FMI) and neighborhood socioeconomic deprivation (SED).

Variable	Model 1. Unadjusted		Model 2. Adjusted ^a^		Model 3. + MVPA ^a^		Model 4. + Sedentary ^a^		Model 5. + Diet ^a^		Model 6. Fully Adjusted ^a^	
	Estimate (SE)		Estimate (SE)		Estimate (SE)		Estimate (SE)		Estimate (SE)		Estimate (SE)	
Neighborhood SED	0.54 (0.19)	**	0.41 (0.20)	*	0.39 (0.21)		0.42 (0.21)	*	0.45 (0.20)	*	0.45 (0.21)	*
MVPA					−0.61 (0.08)	***					−0.45 (0.09)	***
Sedentary							0.20 (0.03)	***			0.12 (0.03)	***
Diet Quality									0.04 (0.02)		0.04 (0.02)	
**Goodness of Fit**												
−2 LL	4488.2		4449.1		4392.1		4407.6		4452.1		4388.3	
AIC	4492.2		4451.1		4396.1		4411.6		4454.1		4392.3	

Note: Neighborhood SED, neighborhood socioeconomic deprivation; MVPA, moderate-to-vigorous physical activity; SE, standard error; −2 LL, log-likelihood; AIC, Akaike’s Information Criterion; * *p* < 0.05; ** *p* < 0.01; *** *p* < 0.001. ^a^ Model adjusted for age, sex, race/ethnicity, parent education, and nesting of students within schools.

**Table 3:** Multilevel logistic regression: odds of overweight/obesity by neighborhood socioeconomic deprivation (SED).

Variable	Model 1. Unadjusted	Model 2. Adjusted ^a^	Model 3. + MVPA ^a^	Model 4. + Sedentary ^a^	Model 5. + Diet ^a^	Model 6: Fully Adjusted ^a^
	OR (95% CI)	OR (95% CI)	OR (95% CI)	OR (95% CI)	OR (95% CI)	OR (95% CI)
Neighborhood SED	**1.36** **(1.10, 1.69)**	**1.29** **(1.03, 1.61)**	**1.28** **(1.01, 1.60)**	**1.29** **(1.02, 1.62)**	**1.31** **(1.05, 1.65)**	**1.30** **(1.03, 1.65)**
MVPA			**0.75** **(0.68, 0.83)**			**0.80** **(0.71, 0.90)**
Sedentary				**1.09** **(1.05, 1.12)**		1.04 (0.99, 1.08)
Diet Quality					1.02 (0.99, 1.05)	1.02 (0.99, 1.05)
**Goodness of Fit**						
−2 LL	1136.2	1132.3	1096.1	1108.4	1130.4	1090.9
AIC	1140.2	1148.3	1114.1	1126.4	1148.4	1114.9

Note: Neighborhood SED, neighborhood socioeconomic deprivation; MVPA, moderate-to-vigorous physical activity; −2 LL, log-likelihood; AIC, Akaike’s Information Criterion; bold typeface indicates significant odds ratio. ^a^ Model adjusted for age, sex, race/ethnicity, parent education, and nesting of students within schools.

## Supplementary Materials

The following are available online at https://www.mdpi.com/1660-4601/17/17/6421/s1, Table S1: Census tract variables used to construct neighborhood socioeconomic deprivation index score; American Community Survey 5 year estimates, 2008–2012; Table S2: Unadjusted and adjusted least squared means of fat mass index among TRACK study participants by neighborhood socioeconomic deprivation (SED) quartiles; Table S3: Odds of overweight/obesity among 5th-grade TRACK study participants by neighborhood socioeconomic deprivation (SED) quartiles.
