# Supplementary material for: Neighborhood Socioeconomic Deprivation Associated with Fat Mass and Weight Status in Youth
Source: Int J Environ Res Public Health. 2020 Sep 3;17(17):6421. doi: 10.3390/ijerph17176421 (PMC7503851; doi:10.3390/ijerph17176421)
Supplement: Supplementary file 1 [file ijerph-17-06421-s001.pdf]

Table S1. Census tract variables used to construct neighborhood socioeconomic deprivation index score; American Community Survey 5-year estimates, 2008–2012.

Domain	ACS Variables	Factor Loading Coefficients ‡	Standardized Scoring Coefficients †
Education	% of total population with less than a high school education *	0.69789	0.13488
Occupation	% of working class *	0.73356	0.14555
	% of civilian labor force unemployed *	0.74070	0.12883
Housing Conditions	% of household ownership	0.40686	−0.02221
	% of vacant households	0.09261	−0.01269
	% of households with more than 1 person per room	0.29262	0.02743
	% of households in poverty *	0.87287	0.11806
	% of female headed households with dependent children *	0.69174	0.07541
	% of households with income <$30,000 *	0.91099	0.13163
	% of households with public assistance *	0.51923	0.10387
	% of households with no car *	0.71970	0.08187
	% of households with no phone *	0.44788	0.05394
	% of households with incomplete plumbing	0.12188	−0.02924
	% of households with no kitchen	0.00341	−0.06318
Income and Poverty	Income disparity *	0.84599	0.13443
	% of population below the federal poverty line *	0.84468	0.10973
Racial Composition	% of population non-Hispanic African American *	0.71688	0.11403
	% of population Hispanic	0.02200	−0.02905
Residential Stability	% of residents aged ≥ 65 years	−0.12764	−0.01385
	% of persons living in same residence for ≥ 5 years	−0.04373	0.08845
	% of foreign born	−0.15830	−0.07625

* Variables selected to generate the deprivation index (Cronbach's alpha = 0.93); ‡ factor loading coefficients (for variables' selection) and † standardized scoring coefficients (for variables' weighting) from the first common factor.

Table S2. Unadjusted and adjusted least squared means of fat mass index among TRACK participants by neighborhood socioeconomic deprivation (SED) quartiles.[1.]

Model	Neighborhood Socioeconomic Deprivation, Quartiles (Q)			
	**Q1 (Affluence)**	**Q2**	**Q3**	**Q4 (Deprivation)**
Unadjusted [2]	5.61 (0.27) [a]	5.74 (0.20) [b]	6.15 (0.26)	6.83 (0.40) [a,b]
Adjusted [3]	5.59 (0.28)	5.68 (0.20)	6.02 (0.27)	6.47 (0.40)
Fully Adjusted [4]	5.64 (0.31) [c]	5.85 (0.24)	6.06 (0.30)	6.64 (0.41) [c]

[1] Model derived estimates presented as adjusted least squared means and standard error (SE); Superscript letters indicate significant differences between adjusted least squared means, $p < 0.05$; [2] Unadjusted model accounted for participants clustered within schools; [3] Model adjusted for age, sex, race/ethnicity, parent education; and accounted for participants clustered within schools; [4] Model adjusted for age, sex, race/ethnicity, parent education, MVPA minutes per hour, sedentary minutes per hour, and diet quality; and accounted for participants clustered within schools. [a] unadjusted model-derived estimate for fat mass index is significantly lower among youth residing in Q1 compared to youth residing in Q4; [b] unadjusted model derived estimate for fat mass index is significantly lower among youth residing in Q2 compared to youth residing in Q4; [c] adjusted model-derived estimate for fat mass index is significantly lower among youth residing in Q1 compared to youth residing in Q4

Table S3. Odds of overweight/obesity among 5th grade TRACK participants by neighborhood socioeconomic deprivation (SED) quartiles.

	Neighborhood SED, Quartiles				
**Models**	**Q1**	**Q2**	**Q3**	**Q4 (dep)**	**Linear Trend**
Model 1. Unadjusted [1]	**0.51 (0.30, 0.86)**	0.73 (0.45, 1.17)	0.82 (0.49, 1.36)	1.0	**<0.05**
Model 2. Adjusted [2]	**0.57 (0.33, 0.97)**	0.74 (0.45, 1.22)	0.80 (0.48, 1.34)	1.0	0.05
Model 3. + MVPA [2]	**0.54 (0.32, 0.95)**	0.70 (0.42, 1.16)	0.70 (0.41, 1.20)	1.0	**<0.05**
Model 4: + Sedentary [2]	**0.53 (0.31, 0.93)**	0.69 (0.42, 1.14)	0.72 (0.42, 1.21)	1.0	**<0.05**
Model 5: + Diet [2]	**0.54 (0.31, 0.93)**	0.73 (0.45, 1.20)	0.79 (0.47, 1.32)	1.0	0.06
Model 6: Fully Adjusted [3]	**0.51 (0.29, 0.90)**	0.67 (0.40, 1.12)	0.67 (0.39, 1.15)	1.0	**<0.05**

[1] Unadjusted model accounted for participants clustered within schools; [2] Model adjusted for age, sex, race/ethnicity, parent education; and accounted for participants clustered within schools; [3] Model adjusted for age, sex, race/ethnicity, parent education, MVPA, sedentary, and diet quality; and accounted for participants clustered within schools. Bold typeface indicates significant odds ratio.
